# Editorial: Veterinary profession in the 21st century – time for a health check

**DOI:** 10.3389/fvets.2023.1338627

**Published:** 2024-01-22

**Authors:** Diana Mendez

**Affiliations:** Australian Institute of Tropical Health and Medicine, Research Division, James Cook University, Townsville, QLD, Australia

**Keywords:** veterinarian, wellbeing, mental health, discrimination, occupational risks

Veterinarians are polyvalent health practitioners who typically work long hours, often in difficult environments and circumstances that can rapidly evolve into crisis scenarios necessitating life-or-death decisions. As such, veterinarians are subjected to a trove of occupational risks that threaten their health and wellbeing (1, 2). They are expected to mitigate these risks while navigating the ethical conundrums of meeting their legal responsibilities (3, 4). Additionally, veterinarians are exposed to more insidious and difficult risks that need to be managed. Overworking, isolation, professional and financial pressures, enacting animal euthanasia, or witnessing animal death or cruelty can lead to psychological distress and in some cases suicide (5, 6). Furthermore, veterinarians must contend with the ever-evolving expectations of their clients, which are often misaligned with the multifaceted roles and responsibilities of veterinarians. The aim of this Research Topic was to elicit contributions that were reflective of the evolving challenges encountered by veterinarians in the 21st century and how these affected their health and wellbeing. This Research Topic includes four original research articles mostly focused on two main stressors experienced by veterinarians: discrimination in the workplace (Berrada et al.; Summers et al.) and difficulties maintaining good veterinarian-clientele relationships (Campbell et al.; Connolly et al.).

In the mid-1990s, French veterinary training institutions experienced the first signs of what was later dubbed “the feminization” of the profession, which has since been observed in many other countries. There were concerns that this change in the veterinary workforce's profile would create gaps in the profession as females were not expected to follow the same career paths into practice as males (7, 8). The introduction of a sex-ratio criterion as part of the entry requirements into French veterinary schools was debated. Although it is now recognized that this approach is unethical, discriminatory, and against entry requirements traditionally based on merit, this trend is still being discussed in the professional French veterinary media. Paradoxically, despite more than 25 years of graduating more female than male veterinarians, the veterinary culture as a whole is still very male-centric (8–10). Furthermore, an examination of the veterinary labor market in France hints at gender-based discrimination being, in part, responsible for a gender wage gap. Included in the current Research Topic, Berrada et al. found that, after controlling for other possible variables, male veterinarians earned wages almost 10% higher than their female counterparts. This is very similar to the gender wage gap reported by the Organization for Economic Co-operation and Development (OECD) for all 27 European Union countries combined between 2018 and 2022 (11). A recent report focused on maternal-based discrimination experienced by female veterinarians in the United States of America, where the gender pay gap across all employees and industries is as high as 17% (11, 12). It is unclear whether these gaps are solely reflective of gender-based discrimination specific to the veterinary industry, a result of differences in career choices and opportunities between male and female veterinarians, or due to broader factors that affect all industries.

Discrimination within the veterinary profession in the United Kingdom (UK) has also been scrutinized, with a third of surveyed veterinarians and veterinary students reporting discriminatory behaviors in the workplace [https://www.bva.co.uk/media/2991/bva-report-on-discrimination-in-the-veterinary-profession.pdf (accessed on 24 October 2023)]. In a follow-up survey by Summers et al., 36% of 403 student participants reported having experienced or witnessed behaviors they perceived as discriminatory in the workplace while completing clinical extramural studies. This survey revealed that most of the behaviors perceived as discriminatory were gender- (38%) and ethnicity-based (15.7%) and were enacted by supervising veterinarians (39.3%) and clientele (36.4%). However, participants reported that most of these incidents, whether experienced or witnessed, went unreported because the concerned parties did not know how to report this type of incident or they were concerned about the consequences. Not addressing these issues may affect staff collaboration in the workplace, the learning experience, and the confidence of students about to enter the workforce and help perpetuate traditional attitudes that are no longer aligned with the widely accepted values of inclusivity and respect and could ultimately have legal repercussions.

People who face multiple forms of discrimination are at a higher risk of experiencing symptoms of depression (13). This pattern may be further compounded among veterinarians who are more likely to experience poor mental health and have suicidal ideations (5). Yet, veterinarians are also known for their lack of mental health help-seeking behaviors. The study by Connolly et al. presented in this Research Topic found that there was a perceived stigma related to poor mental health among Australian and New Zealand veterinarians and that symptomatology indicative of poor mental health, such as burnout, was seen as being normalized within the profession. Additionally, early career veterinarians were more likely to be affected by these factors. The authors concluded that the profession would benefit from building the mental health resilience of current and future veterinarians through undergraduate curriculum design and professional development. Addressing the notoriously poor mental health pervading the veterinary profession would yield positive health and wellbeing outcomes for veterinarians and their colleagues, clients, and patients. In the fourth manuscript in this Research Topic, Campbell et al. interviewed 25 Canadian veterinarians, who thought that working as a veterinarian while experiencing poor mental health could affect workplace interactions with colleagues and clients and alter their ability to concentrate and make sound decisions, which could ultimately affect the quality of care provided to their patients. Perhaps this last key finding could be a powerful health promotion tool if incorporated into mental health self-care and resilience training targeted at current and future veterinarians. By looking after their mental health, veterinarians would not only improve their sense of wellbeing and the workplace dynamics with clients and colleagues, but also improve the welfare and health outcomes of their patients.

This Research Topic provides insights into some of the key challenges the veterinary profession is facing in the 21st century and how these challenges impact mental health. Further research is warranted to identify effective strategies for the profession to overcome these challenges.

## Author contributions

DM: Conceptualization, Writing—original draft, Writing—review & editing.
